# Differentiation of epithelial ovarian cancer subtypes by use of imaging and clinical data: a detailed analysis

**DOI:** 10.1186/s40644-016-0061-9

**Published:** 2016-02-12

**Authors:** Yumiko Oishi Tanaka, Satoshi Okada, Toyomi Satoh, Koji Matsumoto, Akinori Oki, Tsukasa Saida, Hiroyuki Yoshikawa, Manabu Minami

**Affiliations:** Department of Radiology, Faculty of Medicine, University of Tsukuba, 1-1-1 Tennodai, Tsukuba, Ibaraki 305-8575 Japan; Department of Obstetrics and Gynecology, Faculty of Medicine, University of Tsukuba, Tsukuba, 305-8575 Ibaraki Japan; Department of Obstetrics and Gynecology, Tokyo Metropolitan Bokutoh Hospital, Tokyo, 130-8575 Japan

**Keywords:** Ovarian cancer, Serous carcinoma, MRI, CT, Neoadjuvant chemotherapy

## Abstract

**Background:**

Primary epithelial ovarian carcinoma is sub-classified into serous, mucinous, endometrioid and clear cell subtypes. Neoadjuvant chemotherapy has become an alternative treatment option past several years, as serous carcinoma, the most common subtype, is known as chemotherapy-sensitive tumor. On the other hand, mucinous and clear cell carcinoma are known as chemotherapy-resistive. Therefore, it may be meaningful to estimate subtype of ovarian carcinoma using imaging modality. The purpose of this study is to study whether CT or MRI can determine the subtypes of epithelial ovarian cancers.

**Methods:**

The imaging and clinical findings obtained from 125 consecutive patients with primary ovarian carcinoma were retrospectively analyzed. Forty-four of the patients had serous carcinoma; 13, mucinous carcinoma; 53, clear cell carcinoma; and 15, endometrioid carcinoma. We studied the bilateralism, morphological type, tumor diameter, solid portion ratio, relative signal intensity on T2WI and DWI, contrast ratio, and endometriosis on MRI and the calcification, peritoneal dissemination and lymph node metastasis, clinical staging, and thromboembolism on CT. We also studied the tumor markers and serum calcium concentrations. Each parameter was statistically analyzed by univariate and multivariate analyses.

**Results:**

Serous carcinoma showed a significantly higher incidence of bilateral disease, smaller tumor size, higher signal intensity on DWI, and less frequent hypercalcemia. The CA19-9 level was significantly higher in mucinous carcinoma, in which most of the tumors appeared as multilocular cystic masses. Clear cell carcinoma appeared as unilateral disease with a larger solid portion and hypercalcemia in younger patients. Endometrioid carcinoma only showed a lower incidence of intraperitoneal dissemination.

**Conclusions:**

CT and MRI combined with clinical data especially tumor markers and presence of paraneoplastic syndrome could partly predict epithelial ovarian cancer subtypes.

## Background

Primary ovarian tumors are divided into epithelial, mesenchymal, sex cord-stromal, or germ cell origin. Most of malignant ovarian tumors are epithelial origin, which are sub-classified into serous, mucinous, clear cell, and endometrioid carcinomas [1, 2]. The biological behavior of these subtypes and patients’ prognoses, particularly sensitivity to chemotherapy, are quite different from one another [3]. Serous carcinoma (SC) is the most common subtype of epithelial ovarian carcinoma and also known for its sensitivity to platinum-based chemotherapy. On the other hand, the 5-year survival rate of mucinous carcinoma (MC) [3] and clear cell carcinoma (CCC) [4, 5] is significantly poorer than that of serous carcinoma. The current standard treatment for epithelial ovarian carcinoma is primary debulking surgery (PDS) followed by chemotherapy [6, 7]. With this treatment, clinicians can determine the subtype of ovarian carcinoma before starting chemotherapy. In the past several years, however, neoadjuvant chemotherapy (NAC) followed by interval debulking surgery (IDS) has become more commonly performed as an alternative treatment option [8]. With this type of treatment, clinicians often have to start chemotherapy based only upon the cytological diagnosis, in which the pathological subtype of the carcinoma is unclear. However, ineffective NAC may narrow the range of therapeutic window of the patients and physicians prefer to choose PDS for patients with chemo-resistive subtypes. Therefore, it may be meaningful to estimate the subtype of ovarian carcinoma using an imaging modality.

The purpose of this study was to study whether we can determine subtypes of epithelial ovarian cancer by using computed tomography (CT) and magnetic resonance imaging (MRI) with the clinical findings, including the tumor marker and paraneoplastic syndrome such as venous thrombosis or hypercalcemia, to support NAC.

## Methods

### Patient population

**Fig. 1:**
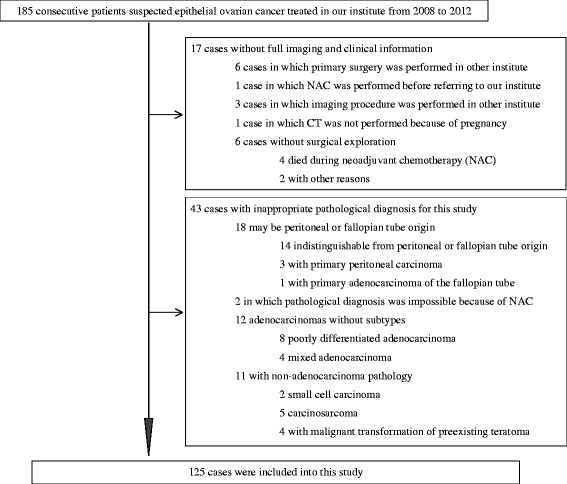
Patient selection. Of the 185 consecutive patients with suspected primary ovarian cancer, 125 were included in this study. The reasons for patient exclusion are presented

This retrospective study was HIPAA-compliant and approved by the Ethics Committee of Tsukuba University Hospital with a waiver of documentation of written informed consent (H25-63). From January 2008 through December 2012, 185 consecutive patients with suspected primary ovarian cancer were treated at our institute. Seventeen cases with insufficient imaging or clinical information and 43 cases with inappropriate pathological diagnosis for this study were excluded (Fig. 1). Therefore, 125 cases (aged 31–85 years, mean age 59 years) were included in the study. One hundred two patients were treated with PDS followed by chemotherapy and 23, with NAC followed by IDS. The number of patients with SC, MC, CCC, and endometrioid carcinomas (EC) were 44, 13, 53 and 15, respectively (Table 1).

**Table 1:** Patients Population

Histopathologic Subtypes	Age (years old)	Treatment		Total Number of Cases
		PDS	NAC	
Serous	61 (31–85)	24	20	44
Mucinous	59 (37–84)	12	1	13
Clear cell	57 (37–81)	51	2	53
Endometrioid	59 (43–84)	15	0	15
Total	59 (31–85)	102	23	125

Final objectives are shown with histopathological subtypes, age and treatment

### CT and MRI examinations

Contrast CT and MRI were conducted in all cases. The CT images were acquired at 120 kVp and between 150 and 400 mAs and were reconstructed at 2- and 5-mm intervals with a 16 or 64 multidetector-row scanner (Brilliance 16 or Brilliance 64; Philips Medical Systems, Best, the Netherlands). All patients were given 800 mL of diluted oral contrast material (sodium amidotrizoate and meglumine amidotrizoate [Gastrografin; Bayer Health Care, Berlin, Germany]) 1 h before the scan. Noncontrast CT covered the entire primary lesion in the adnexa and the contrast study covered the whole abdomen and pelvis. Contrast-enhanced CT images were obtained after the injection of 100 mL of a nonionic iodinated contrast material with a concentration of 370 mg/mL (iopamidol [Iopamiron 370; Bayer Health Care]) or 300 mg/mL (iohexol [Omnipaque 300; Daiichi-Sankyo Health Care, Tokyo, Japan]) or 240 mg/mL (ioversol [Optilay 240; Covidien, Dublin, Ireland]) at a rate of 2 mL/s. Equivalent phase images were obtained during at least one 50-s delay.

MRI was obtained with 1.5 T superconducting units (Intera or Achieva; Philips Medical Systems). An intramuscular injection of hyoscine butylbromide (Buscopan; Boehringer Ingelheim, Ingelheim, Germany) was given to all patients to reduce bowel peristalsis. The MRI protocol, including spin-echo T1-weighted imaging (T1WI), fast spin-echo T2-weighted imaging (T2WI), fat-saturated T1-weighted imaging, contrast-enhanced and fat saturated spin-echo T1-weighted imaging with 5 mmoL of gadopentetate dimeglumine (Magnevist; Bayer Health Care), was obtained in all cases. Additional echo-planar diffusion-weighted images (DWI) were obtained in 98 cases. All MRIs were obtained with a slice thickness of 4 to 6 mm, an intersection gap of 0.4 to 0.6 mm, a matrix of 320 x 320 – 512 x 512, a field of view (FOV) of 28 to 36 cm, and 2 to 4 excitations. The other MRI parameters were as follows: (a) repetition time (TR)/echo time (TE) 53–575/11–14 msec for spin-echo T1WI; (b) TR/TE 1600–2100/100 msec, 16-echo train length for fast spin-echo T2WI; (c) TR/TE 600–650/10–13 msec for fat-saturated T1WI with spectral presaturation with inversion recovery; (d) TR/TE 5000/55 msec, b-value 1000 s/mm^2^, 19-echo train length for DWI.

### Image analysis

All the images were retrospectively reviewed by 2 trained radiologists with more than 10 years of experience in gynecological MRI. For cases in which the radiologists’ opinions differed, consensus extents were allocated. We analyzed the nature of the primary lesion: its morphological type, unilateral or bilateral nature, maximum diameter of the larger adnexal mass, maximum diameter of the largest mural nodule, ratio of the solid to cystic portion, signal intensity of the solid portion on T2WI compared with the skeletal muscles, signal intensity of the solid part on DWI compared with the endometrium, and intratumoral calcification. Morphological types were divided into 4 types by visual inspection: multilocular cystic, unilocular cyst with a solitary mural nodule, unilocular cyst with multiple mural nodules, and multilocular cysts with a solid or predominantly solid portion. The degree of calcification was classified into none, a few, and multiple. The ancillary findings were also noted: intraperitoneal dissemination, lymph node metastasis, preoperative staging diagnosis with CT and MRI, coexistent endometriosis, and venous thrombosis. On the basis of previous research [9, 10] and our own experience, lymph node metastasis was considered positive if either of the following was shown: solitary lymph node larger than 10 mm in minimal diameter or clustered lymph nodes larger than 6 mm in minimal diameter. The endometriosis was considered positive when a multilocular cystic adnexal mass with hemorrhagic contents and a thick capsule or septa or adhesion in the pelvic floor was noted [11, 12]. Venous thrombosis was considered positive if a filling defect was present within the iliac veins or IVC on contrast-enhanced CT.

### Clinical information

Clinical information including serum levels of CA125, CA19-9, CEA, and calcium was collected by retrospective review of the medical records. Serum calcium levels of 10.4 mg/dl or higher were considered to indicate hypercalcemia.

### Pathological diagnosis

Pathological diagnosis was done depending on the official pathological reports of our institute including the subtypes of ovarian cancer, histopathological stage, and coexisting pathology such as endometriosis.

### Statistical analysis

The data were analyzed by both univariate and multivariate analyses. For the univariate analysis, all of the parametric values, including age, largest tumor diameter, largest solid portion diameter, ratio of the solid portion, T2 signal ratio, DWI signal ratio, contrast ratio, and levels of serum CA125, CA19-9, and CEA, were analyzed using one-way variance of analysis (ANOVA) and the Bonferroni multiple comparison test as the post hoc test. The nonparametric values, including bilaterality, morphology, calcification, dissemination, lymph node metastasis, staging, endometriosis, and thrombosis, were analyzed using the Kruskal-Wallis test and the Dunn multiple comparison test as the post hoc test (Prism 5 for Mac OS X; Graphpad Software, La Jolla, CA, USA). Multivariate analysis was performed with binary logistic regression (SPSS Statistics; IBM, New York, NY, USA).

## Results and discussion

The results for each parameter, the univariate analysis, and the multivariate analysis are listed in Tables 2, 3, and 4, respectively.

**Table 2:** The Result of the each parameter in each subtype

		Serous	Mucinous	Clear cell	Endometrioid
Number of Patients		44	13	53	15
Age (mean)		61	59	57	59
Largest tumor diameter (mean, mm)		79.5	193.3	138.328	126.9
Largest solid part diameter (mean, mm)		50.4	24.6	63.3	57.2
Ratio of the solid part (mean)		0.70	0.14	0.48	0.47
T2 signal ratio (mean)		4.16	5.30	4.86	4.08
DWI signal ratio (mean)		1.84	1.18	1.37	1.56
Contrast Ratio (mean)		0.84	1.18	1.14	0.92
Bilaterality	Unilateral	13	11	48	14
	Bilateral	31	2	5	1
Morphology	Multilocular cystic	1	6	0	0
	Unilocular with single nodule	4	1	6	2
	Unilocular with multiple nodule	1	0	16	5
	Multilocular with solid	17	6	28	8
	Solid	21	0	3	0
Calcification	-	41	10	42	12
	+	3	3	11	3
Dissemination	-	11	8	38	13
	+	3	0	1	1
	++	30	5	14	1
LNs mets	-	20	9	40	11
	+	15	4	9	3
	++	9	0	4	1
Staging	I	2	5	22	5
	II	2	1	9	5
	III	25	5	15	4
	IV	15	2	7	1
Endometriosis	-	36	11	32	6
	+	8	2	21	9
Thrombosis	-	44	13	50	15
	+	0	0	3	0
CA125 (mean, U/ml)		2081.6	225.6	683.6	1936.5
CA19-9 (mean, U/ml)		711.9	13899.0	926.4	352.0
CEA (mean, ng/ml)		1.87	33.91	3.08	16.49
Hypercalcemia	-	43	12	39	14
	+	1	1	13	1

The result of each parameter is tabulated in this table

**Table 3:** The results of univariate analysis

	All 4 subtypes	Serous vs Mucinous	Serous vs Clear cell	Serous vs Endometrioid	Mucinous vs Clear cell	Mucinous vs Endometrioid	Clear cell vs Endometrioid
	*P* value	*P* < 0.05 ?	*P* < 0.05 ?	*P* < 0.05 ?	*P* < 0.05 ?	*P* < 0.05 ?	*P* < 0.05 ?
Age	N.S.	N.S.	N.S.	N.S.	N.S.	N.S.	N.S.
Largest tumor diameter	<0.0001	^***^	^***^	^*^	N.S.	^*^	N.S.
Largest solid part diameter	0.0016	N.S.	N.S.	N.S.	***	N.S.	N.S.
Ratio of the solid part	<0.0001	^***^	^**^	N.S.	^**^	^*^	N.S.
T2 signal ratio	N.S.	N.S.	N.S.	N.S.	N.S.	N.S.	N.S.
DWI signal ratio	0.0100	N.S.	^**^	N.S.	N.S.	N.S.	N.S.
Contrast Ratio	0.0263	N.S.	N.S.	N.S.	N.S.	N.S.	N.S.
Bilaterality	<0.0001	^**^	^***^	^***^	N.S.	N.S.	N.S.
Morphology	<0.0001	^***^	^***^	^**^	N.S.	N.S.	N.S.
Calcification	0.2328	N.S.	N.S.	N.S.	N.S.	N.S.	N.S.
Dissemination	<0.0001	N.S.	^***^	^***^	N.S.	N.S.	N.S.
LNs mets	0.0116	N.S.	^*^	N.S.	N.S.	N.S.	N.S.
Staging	<0.0001	N.S.	^***^	^**^	N.S.	N.S.	N.S.
Endometriosis	0.0051	N.S.	N.S.	^*^	N.S.	N.S.	N.S.
Thrombosis	0.2465	N.S.	N.S.	N.S.	N.S.	N.S.	N.S.
CA125	0.0502	N.S.	N.S.	N.S.	N.S.	N.S.	N.S.
CA19-9	0.0003	^***^	N.S.	N.S.	^***^	^*^	N.S.
CEA	<0.0001	^*^	N.S.	N.S.	^*^	N.S.	N.S.
Hypercalcemia	0.0096	N.S.	^**^	N.S.	N.S.	N.S.	N.S.

Parametric factors were analyzed with one-way ANOVA, followed by a Bonferroni multiple comparison test was applied for a post hoc -test

Nonparametric factors were analyzed with the Kruskal-Wallis test, followed by a Dunn post hoc test

^*^significant with low *p* values, ^**^ significant with lower *p* values, and ^***^significant with the lowest *p* values

**Table 4:** The results of multivariate analysis

		B	*P* value	Odd’s ratio	95 % CI of OR
Serous adenocarcinoma	Age	.086	.040	1.090	1.004–1.184
	Largest tumor diameter	−.038	.001	.963	.942–.984
	DWI signal ratio	1.544	.016	4.684	1.329–16.510
	Bilaterality	2.952	.001	19.146	3.149–116.424
	Hypercalcemia	−3.804	.013	.022	.001–.452
Mucinous adenocarcinoma	Ratio of the solid part	−11.603	.039	.000	.000–.562
	Calcification	1.487	.071	4.425	.882–22.195
	CA19-9	.002	.009	1.002	1.000–1.003
Clear cell adenocarcinoma	Age	−.099	.002	.906	.850–965
	Largest solid part diameter	.021	.031	1.021	1.002–1.040
	Bilaterality	−3.000	.000	.050	.010–.258
	Hypercalcemia	2.709	.011	15.009	1.859–121.174
Endometrioid adenocarcinoma	Dissemination	−.916	.051	.400	.160–1.003

Multivariate analysis was performed using binary logistic regression. The tables indicate only independent variables that showed multicollinearity for each subtype

**Fig. 2:**
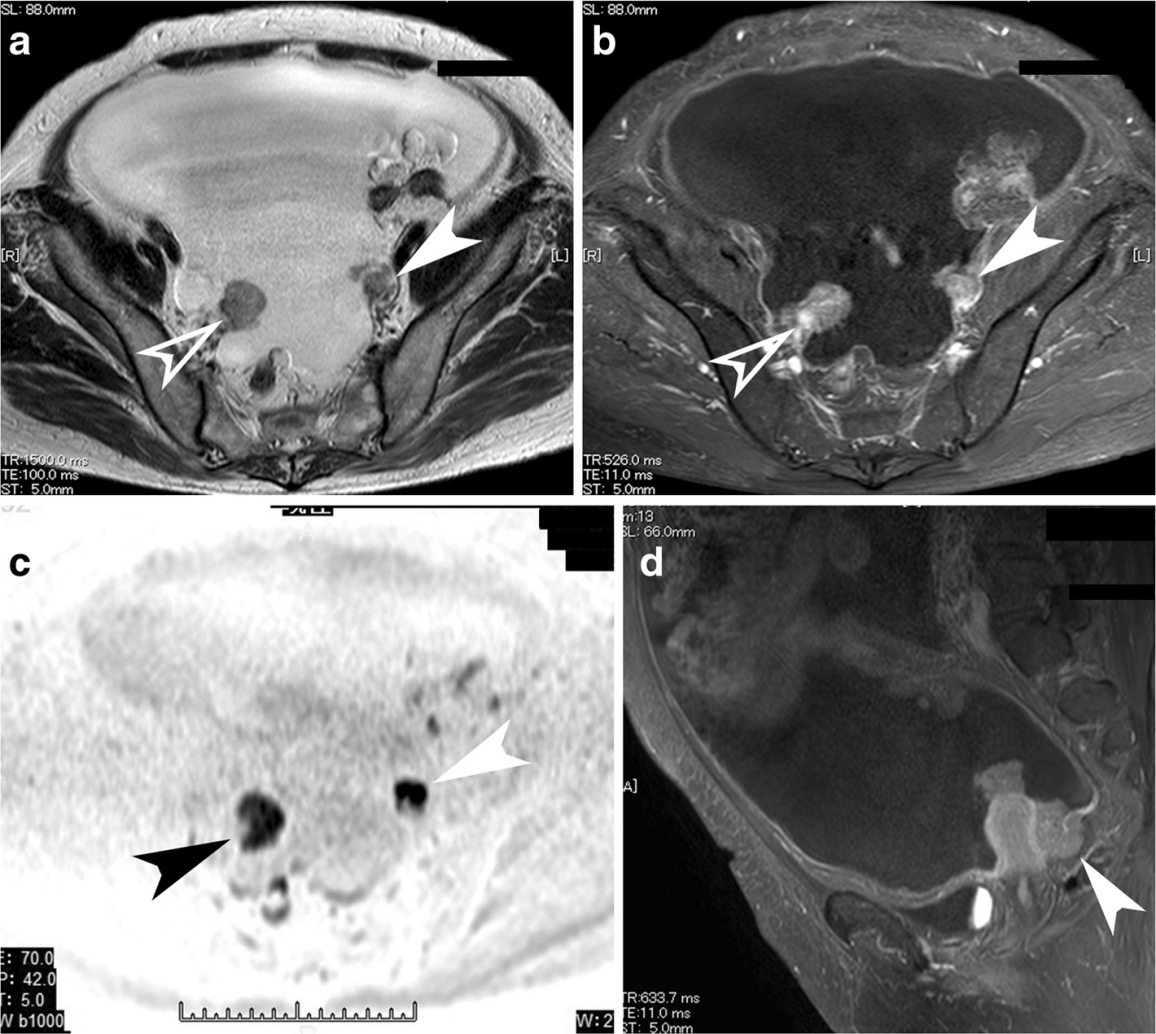
A case with serous carcinoma. Small almost entirely solid small masses are replacing the bilateral ovaries (arrows, **a**: axial T2-weighted image, **b**: axial contrast-enhanced and fat-saturated T1-weighted image). Both masses show strong restricted diffusion on diffusion-weighted image (**c**, arrowheads). Note the massive ascites and thickened parietal peritoneum with a mass in the cul-de-sac (arrowhead) on sagittal and contrast-enhanced and fat-saturated T1-weighted image (**d**), reflecting the extensive peritoneal dissemination

Serous carcinoma showed a strong prediction for bilateral disease (*p* = .040, multivariate analysis; the same shall apply hereinafter), smaller tumor size (*p* = .001), and restricted diffusion (*p* = .016), especially when compared with CCC. It also tended to appear as predominantly solid masses, although this difference was not significant on multivariate analysis (Fig. 2). In addition, hypercalcemia was observed significantly less often (*p* = .013).

**Fig. 3:**
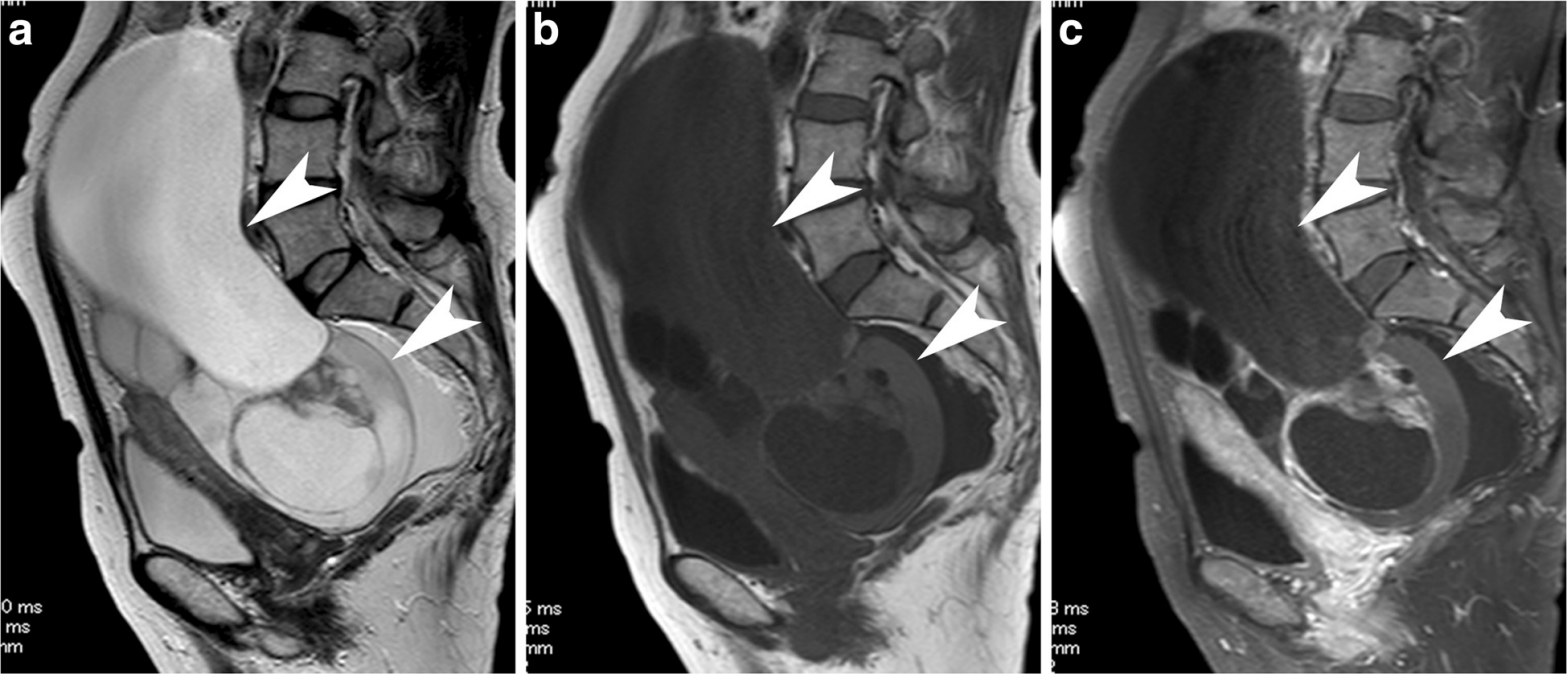
A case with mucinous carcinoma. A multilocular cystic mass with varying signal intensity, so called stained-glass tumor, is seen on sagittal T2-weighted image (arrow heads, **a**). After administration of contrast material, we can see some small solid parts along the septa enhanced by contrast material (arrow heads, **b**: sagittal T1-weighted image, **c**: contrast-enhanced and fat-saturated T1-weighted image)

CA19-9 levels were significantly higher in MC (*p* = .009). Twelve of the 13 MC appeared as multilocular cystic masses, although this difference was not statistically significant and only the smaller ratio of the solid portion was evident (*p* = .039) (Fig. 3).

**Fig. 4:**
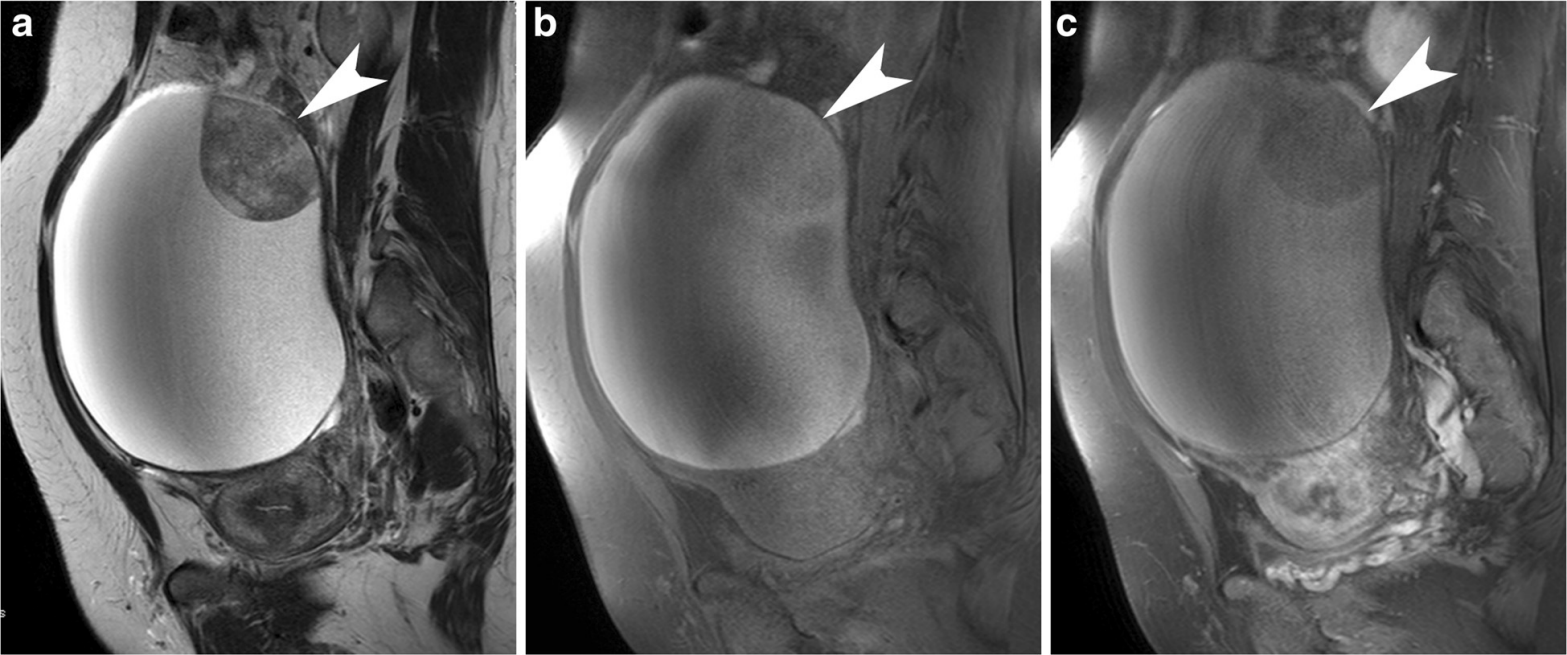
A case with clear cell carcinoma. A solitary large unilocular cystic mass with a mural nodule is demonstrated on the uterus on sagittal T2-weighted image (**a**). The lack of adipose tissue between the mass and the uterus suggest adhesion, which evoke co-existing endometriosis. In addition, the content of the cyst shows high signal intensity on sagittal fat-saturated T1-weighted image is another evidence of hemorrhagic material included within an endometriotic cyst (**b**). After administration of contrast material, the mural nodule is weakly enhanced (arrow heads, b: sagittal T1-weighted image, **c**: contrast-enhanced and fat-saturated T1-weighted image)

Clear cell carcinoma tended to appear as unilateral disease (*p* = .000) with a larger solid portion (*p* = .031) in younger patients (*p* = .002). Hypercalcemia was also commonly seen in CCC (*p* = .011, Fig. 4).

**Fig. 5:**
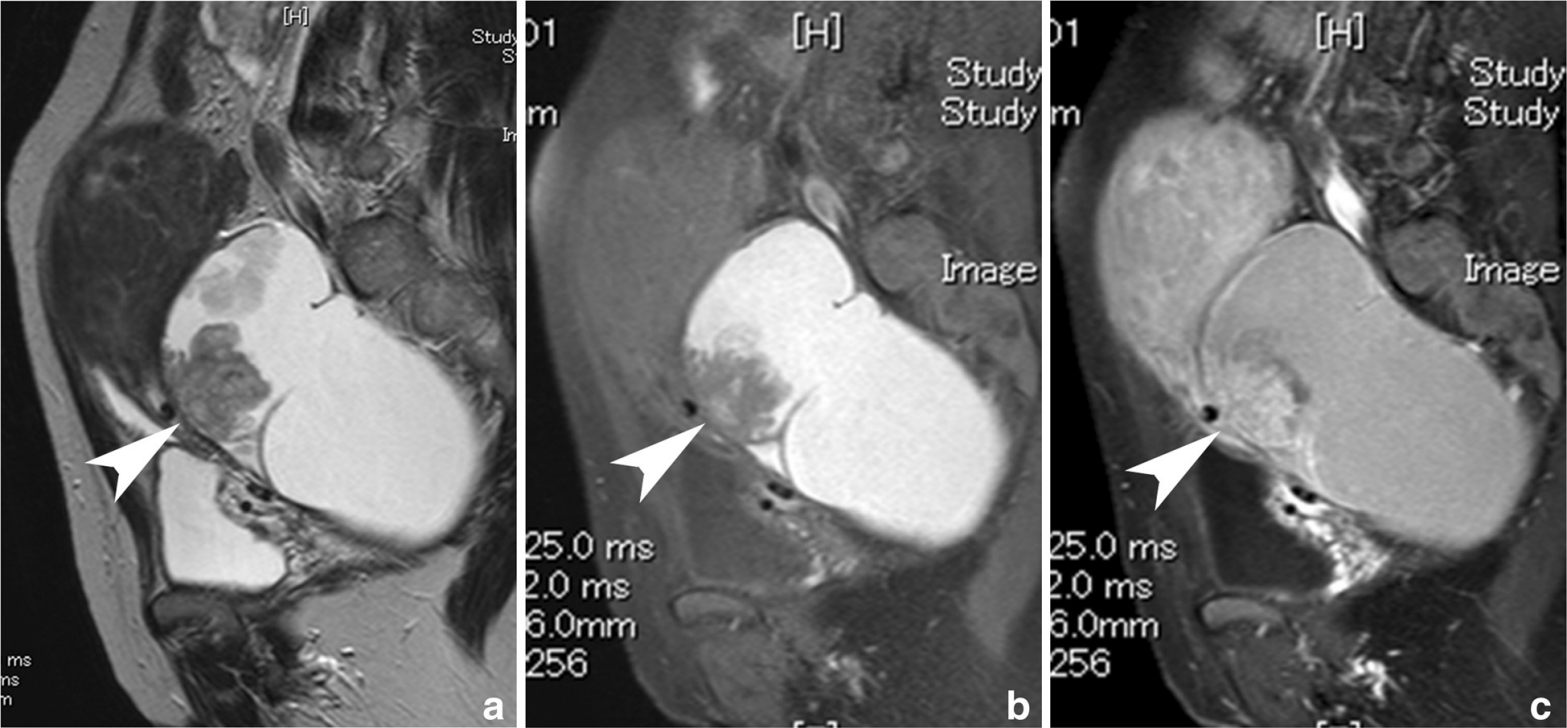
A case with endometrioid carcinoma. A multilocular cystic mass with mural nodules is seen in the cul-de-sac adhering to the uterus on sagittal T2-weighted image (a). The content of the cyst also shows high signal intensity on sagittal fat-saturated T1-weighted image  (b), suggesting co-existing endometriosis. The mural nodules are also well enhanced (arrow heads, c: contrast-enhanced and fat-saturated T1-weighted image). As these imaging characteristics share those of serous and clear cell carcinoma, the specific diagnosis with imaging findings seems the most difficult among 4 subtypes

Only intraperitoneal dissemination was significantly less common in EC (*p* = .051). CCC and EC are subtypes well known as complicated by endometriosis (Fig. 5) [13, 14]; however, in this study neither subtype showed any significant increase in endometriosis in the multivariate analysis.

Primary epithelial ovarian tumors are sub-classified into serous, mucinous, clear cell, and endometrioid carcinomas [1, 2]. Primary debulking surgery followed by chemotherapy is an established standard treatment for epithelial ovarian carcinoma, having achieved excellent therapeutic results with the use of cytotoxic agents [6, 7]. These excellent results may be due to the fact that the vast majority of ovarian carcinoma are of the serous subtype, particularly in the United States and Europe. On the other hand, in the past couple of decades, the number of primary ovarian cancers accompanied by endometriosis has rapidly increased [13, 15]. It is well known that CCC and EC are common subtypes arising from ovarian endometriotic cysts [13, 15]. As the incidence of ovarian cancer accompanied by endometriosis has become higher, the incidence of CCC has become much higher in Japan than in the United States and Europe [15]. CCC is also known as a chemotherapy-resistant subtype of ovarian carcinoma [4, 5]. Therefore, clinicians want to avoid NAC in patients with this subtype.

Although the imaging findings of ovarian carcinoma have been rarely reported, we can speculate on its morphological characteristics on the basis of the macroscopic findings reported in the pathology literature. Serous carcinoma has been characterized by psammomatous calcification on CT [16], peritoneal carcinomatosis, relatively small ovaries, and a highly elevated serum CA-125 level [17]. Our findings revealed that bilateral disease with a smaller tumor size is also common in SC; however, calcification and intraperitoneal dissemination were not significantly frequent in our results. It has also been reported that intratumoral calcification may be observed in ovarian carcinomas other than SC [18] and that on CT psammomatous calcification could not be differentiated from other calcifications [19]. Therefore, our findings may not be surprising. Recently, SC has been subdivided into low-grade and high-grade subtypes [20]. Low-grade serous carcinoma (LGSC) is considered to arise from serous borderline tumors. Serous surface papillary borderline tumors (SSPBTs) are characterized by a papillary architecture and internal branching pattern, like that of a sea anemone [21]. Therefore, LGSC may resemble SSPBTs that tend to make larger tumors. Our findings might have included a few LGSCs, as the incidence of the LGSC has been reported as far lower than those of high-grade serous carcinomas [22]. We speculate that this inhomogeneity in the SC group might have influenced the present findings.

Mucinous carcinoma is also a chemotherapy-resistant subtype of ovarian carcinoma [3]. MRI findings characteristic of MC have been reported as a large multilocular cystic mass with varying signals [23, 24], and its so-called stained glass-like appearance may be a hallmark of the tumor. In this study, we classified the morphological subtypes into 5 categories, and 12 of the 13 MC showed multilocular cystic with and without a solid portion. However, we could not reveal that the multilocular cystic mass was characteristic of MC because many of the cases of the other subtypes also had a multilocular cystic morphology with solid masses. The former WHO classification of ovarian tumors classified MC into intestinal and endocervical-like subtypes [1]. In the latest version of the WHO classification, endocervical-like MC is defined as seromucinous carcinoma [2]. However, our study was performed following the former version of the WHO classification [1] and thus included endocervical-like MC. Endocervical-like MC usually appears as mural nodules of endometriotic cysts [2]. Our results also revealed a high incidence of endometriosis in MC. This high incidence might suggest that endocervical-like MC is difficult to differentiate from CCC or EC.

Clear cell carcinoma and endometrioid carcinoma are commonly complicated by endometriosis [13, 15]. Therefore, coexistent endometriosis may be the key finding for these subtypes [14, 25, 26]. In our study, however, only CCC showed a significantly higher incidence of endometriosis when compared with SC in the univariate analysis. In addition, CCC was reported to appear as a larger unilocular cystic mass with eccentric mural nodules [27, 28], whereas in our study a large number of CCC had multilocular cystic masses. Therefore, differentiating CCC from EC based only on the imaging findings seems to be difficult. On the other hand, as in previous reports, coexistent hypercalcemia was more commonly seen in CCC in our study [29], which may be helpful for the differential diagnosis. Venous thrombosis was also reported as a frequently seen paraneoplastic syndrome of CCC [30]; however, our study could only reveal that the incidence of venous thrombosis was lower in SC.

Endometrioid carcinoma, a chemosensitive subtype of ovarian carcinoma, only showed a significantly lower incidence of peritoneal dissemination. In other words, it did not show any unique characteristics in its morphology.

Some limitations in our study should be pointed out. First, we could analyze only the morphological characteristics of the ovarian carcinomas, whereas numerous histological types of tumors affect the ovaries. Therefore, the process of differential diagnosis in daily practice may be more complex. Second, the number of tumors that we analyzed was limited. We included relatively larger number of CCC as our study population included a lot of ovarian cancer coexisting endometriosis. However, a bigger number of tumors need to be analyzed, in particular MC and EC.

## Conclusions

In conclusion, ovarian serous carcinoma tended to appear as smaller, bilateral masses with more highly restricted diffusion without hypercalcemia.

### Availability of supporting data

The data sets supporting the results of this article are included within the article.

## Abbreviations

ANOVA: one-way variance of analysis (ANOVA)
CCC: clear cell carcinoma
CT: computed tomography
EC: endometrioid carcinomas
IDS: interval debulking surgery
MC: mucinous carcinoma
MRI: magnetic resonance imaging
NAC: neoadjuvant chemotherapy
PDS: primary debulking surgery
SC: serous carcinoma
TE: echo time
TR: repetition time
